# Tumor repolarization by an advanced liposomal drug delivery system provides a potent new approach for chemo-immunotherapy

**DOI:** 10.1126/sciadv.aba5628

**Published:** 2020-09-04

**Authors:** L. Ringgaard, F. Melander, R. Eliasen, J. R. Henriksen, R. I. Jølck, T. B. Engel, M. Bak, F. P. Fliedner, K. Kristensen, D. R. Elema, A. Kjaer, A. E. Hansen, T. L. Andresen

**Affiliations:** 1 Department of Health Technology, Biotherapeutic Engineering and Drug Targeting, Technical University of Denmark, Kgs. Lyngby, Denmark.; 2 Department of Clinical Physiology, Nuclear Medicine and PET and Cluster for Molecular Imaging, Department of Biomedical Sciences, Rigshospitalet and University of Copenhagen, Copenhagen, Denmark.; 3 The Hevesy Laboratory, Center for Nuclear Technologies, Technical University of Denmark, Roskilde, Denmark.

## Abstract

Immunosuppressive cells in the tumor microenvironment allow cancer cells to escape immune recognition and support cancer progression and dissemination. To improve therapeutic efficacy, we designed a liposomal oxaliplatin formulation (PCL8-U75) that elicits cytotoxic effects toward both cancer and immunosuppressive cells via protease-mediated, intratumoral liposome activation. The PCL8-U75 liposomes displayed superior therapeutic efficacy across all syngeneic cancer models in comparison to free-drug and liposomal controls. The PCL8-U75 depleted myeloid-derived suppressor cells and tumor-associated macrophages in the tumor microenvironment. The combination of improved cancer cell cytotoxicity and depletion of immunosuppressive populations of immune cells is attractive for combination with immune-activating therapy. Combining the PCL8-U75 liposomes with a TLR7 agonist induced immunological rejection of established tumors. This combination therapy increased intratumoral numbers of cancer antigen–specific cytotoxic T cells and Foxp3^−^ T helper cells. These results are encouraging toward advancing liposomal drug delivery systems with anticancer and immune-modulating properties into clinical cancer therapy.

## Teaser

Liposomal oxaliplatin remodels the tumor microenvironment and potentiate TLR-agonist–mediated immune responses in cancer.

## INTRODUCTION

Cancer cells have effective immunoediting abilities that are central for establishing the immunosuppressive tumor microenvironment (TME). These properties allow cancer cells to escape immune recognition and support the cancer progression and dissemination (*1*). The TME comprises a sophisticated interplay between cancer cells, stromal cells, and infiltrating immune cells, and the immune cell composition is directly correlated with therapeutic prognosis across current anticancer therapies (*2*, *3*). Several conventional chemotherapeutics and radiation therapy have been identified to have potent immune-stimulating properties through induction of immunogenic cell death (ICD) (*4*, *5*). Considering that liposomal drug delivery systems are adjustable in terms of circulatory properties, organ targeting, and cellular interactions, we hypothesized that optimized drug delivery vehicles would improve cancer cell cytotoxicity and immunomodulation.

**Fig. 1:**
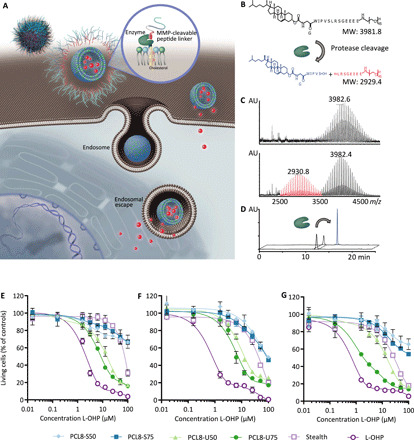
Cleavable liposomes provide improved cellular interaction and in vitro cytotoxicity. (**A**) Schematic illustration of protease-mediated liposome dePEGylation in the TME and subsequent uptake by cells. The structure of PCL and the MMP cleavage site is displayed in (**B**) where proteolysis will create two fragments as illustrated by matrix-assisted laser desorption/ionization–time-of-flight (MALDI-TOF) (**C**) and high-performance liquid chromatography (HPLC) (**D**). MW, molecular weight; AU, arbitrary units.; *m/z*, mass/charge ratio. Investigation of optimal PCL oxaliplatin (L-OHP) liposome composition for killing cancer cells in vitro including the comparison to stealth L-OHP liposomes and free L-OHP. Liposome containing 8% PCL and 5% (S50) or 7.5% (S75) cationic saturated lipids or 5% (U50) or 7.5% (U75) unsaturated cationic lipids. (**E**) CT26 (murine colorectal cancer), (**F**) 4T1 (murine breast cancer), and (**G**) B16.F10 (murine melanoma).

Several therapeutic targets beside the cancer cells exist in the TME. monocytic myeloid-derived suppressor cells (Mo-MDSCs) represent a central immunosuppressive population with potent protumorigenic properties and inhibitory activities on cytotoxic (cT) T cells and natural killer cell effector function (*6*–*8*). Furthermore, they are progenitors to protumorigenic tumor-associated macrophages (TAMs) (*9*, *10*). Increased tumor levels of Mo-MDSCs are related to poor prognosis, and depleting these from the TME augments the effect of immunotherapies (*11*–*13*). Mo-MDSCs have a lower phagocytic capacity than the immunosuppressive TAMs and patrolling monocytes (*14*). Consequently, it is challenging to achieve sufficient drug targeting and activity to deplete this important cell population. We therefore hypothesized that improved bioavailability of oxaliplatin (L-OHP) in Mo-MDSCs would be achieved if sufficient concentrations of cationic liposomes were delivered to the TME considering their capabilities for inducing high cell uptake and as transfection vectors (*15*, *16*). Such delivery systems may potentially improve intracellular drug trafficking and allow it to reach cytotoxic concentrations at the site of action, which may not be achievable via phagocytosis in Mo-MDSCs alone. Historically, liposomal drug delivery systems have focused on optimizing tumor accumulation via the enhanced permeability and retention (EPR) effect by securing prolonged circulating properties of liposomes (*17*, *18*). This is still indispensable for tumor accumulation, but too stable liposomes have been identified to have slow drug release and limited drug availability in the tumor (*19*). The ideal liposome formulation aims at achieving high, EPR-mediated, intratumoral liposome accumulation and effectively delivers therapeutic cargo. While polyethylene glycol (PEG) greatly enhances the circulatory stability of the liposomes, it hinders effective drug release in the tumor. PEGylation interferes with cellular internalization and intracellular endosomal liposome trafficking and escape, which reduce bioavailability of L-OHP (*20*). To circumvent these problems, we developed a PEGylated liposome system that, upon accumulation in tumors, shed the PEG layer when exposed to endogenous levels of intratumoral proteases (Fig. 1A). Proteases, such as matrix metalloproteinases (MMPs), are highly expressed across solid cancers and play major roles in tumorigenesis (*21*). The shedding of the anionic PEG-glutamic acid layer exposes a more cationic particle for improved interaction and toxicity against both cancer cells and the challenging Mo-MDSC population.

L-OHP is among the most potent inducers of ICD. It triggers pre-apoptotic exposure of calreticulin and extracellular release of ATP and high-mobility group box 1 (HMGB1) (*5*, *22*, *23*). The released factors have potent immune-activating properties, including Toll-like receptor 4 (TLR4) activity (*24*). The potential of L-OHP to kill cancer cells and induce immune activation makes it an interesting candidate. The presented data illustrate that EPR-dependent tumor accumulation can be achieved using a shielded cationic lipid L-OHP liposome formulation. The demonstrated anticancer properties of the designed liposomal drug delivery system include direct cancer cell cytotoxicity and depletion of immunosuppressive populations, which induce an immune-mediated anticancer activity in syngeneic cancer models.

## RESULTS

### Cytotoxicity and EPR-dependent tumor accumulation

PEGylation of liposomes not only is essential to achieve long systemic circulation of liposomes but also impedes drug release, depending on liposome composition and cell interactions (*20*, *25*). To overcome this, we constructed a liposomal L-OHP drug delivery system based on a cationic unsaturated lipid composition shielded with a negatively charged, PEGylated, protease cleavable lipopeptide. This vehicle maintains circulation characteristics of stealth liposomes and provides means to achieve improved intratumoral drug release upon accumulation in solid cancers.

Upon intratumoral accumulation, proteases expressed in the TME cleaves the PEGylated cleavable lipopeptide (PCL) peptide motif and thereby dePEGylates and destabilizes the liposome, which improves cell interactions and drug release (Fig. 1, A to D). The used PCL construct is anchored to the liposomal membrane by cholesterol and contains four negatively charged glutamic acid residues as part of the protease-cleavable motif (Fig. 1B) (*26*). During systemic circulation, the liposome is PEGylated, and the liposome charge is balanced by the PCL due to its anionic charge. Different L-OHP liposome formulations composed of either unsaturated [1-palmitoyl-2-oleoyl-*sn*-glycero-3-phosphocholine (POPC) and 1,2-dioleoyl-3-trimethylammonium-propane (DOTAP)] or saturated [1,2-distearoyl-*sn*-glycero-3-phosphocholine (DSPC) and 1,2-stearoyl-3-trimethylammonium-propane (DSTAP)] lipids and varying cationic charge (5 or 7.5%) were investigated by in vitro cytotoxicity across three cancer cell lines, CT26 (murine colorectal cancer), B16.F10 (murine malignant melanoma), and 4T1 (murine breast cancer), using PEGylated stealth liposomes [l-a-phosphatidylcholine, hydrogenated (Soy) (HSPC):cholesterol:1,2-distearoyl-sn-glycero-3-phosphocholine (DSPE)–PEG2000 57:38:5 molar ratio] and free L-OHP as controls (Fig. 1, E to G). L-OHP displayed, as expected, the most potent in vitro effect, while the stable stealth liposomes displayed low toxicity even at high concentrations, suggesting low spontaneous leakage and low cellular activity. The PCL-functionalized liposomes resulted in improved cytotoxicity compared to stealth liposomes in all cell lines tested, and the most optimal composition in terms of potency was the PCL8-U75 liposomes containing 8% PCL and 7.5% unsaturated cationic lipids (POPC:Cholesterol:DOTAP:PCL 52.5:32.0:7.5:8.0 molar ratio; Fig. 1, E to G).

Pharmacokinetics and biodistribution of the four different PCL formulations were investigated in tumor-bearing NMRI (Naval Medical Research Insititute) nude mice and compared with stealth and free L-OHP (fig. S1, A and B). Here, we observed that the saturated formulation containing 7.5% cationic lipids had a shorter circulating half-life than the other formulations and accumulated, to a larger extent, in the liver and spleen. Consequently, tumor accumulation was reduced compared to the other formulations. Both unsaturated formulations had a more favorable biodistribution than the saturated formulations. Pharmacokinetics of a saturated formulation containing 5% PCL (PCL5-S75) was also tested, and the results demonstrated that inclusion of only 5% PCL in the membrane was not sufficient to shield the liposomes and secure circulating properties (fig. S1, C and D). Compared to stealth liposomes, this formulation was rapidly cleared, and after 24 hours, we could detect increased amounts of platinum in the liver and spleen. Because we wanted to include a high cationic lipid concentration that was still able to circulate similar to stealth liposomes, the PCL8-U75 formulation was chosen as our leading candidate. In addition, we confirmed ex vivo that cleaving the PEG layer from both PCL8-U75 and PCL8-U50 improved the uptake by cancer cells significantly (fig. S1E). Figure S2 table 1 describes the characteristics of the formulations used in this study. To ensure that the formulated L-OHP–containing liposomes were monodisperse, homogeneous, and of the expected size, we performed cryo–transmission electron microscopy on the optimal candidate formulation PCL8-U75 and PCL8-S75 both prepared by hydration of freeze-dried lipids (fig. S3, A and B, respectively). The liposomes had well-defined membranes and an average size of around 100 nm. PCL8-U75 with encapsulated L-OHP was stable for 6 months at 4°C with respect to size, zeta potential, and L-OHP retention (fig. s2 table 2).

**Fig. 2:**
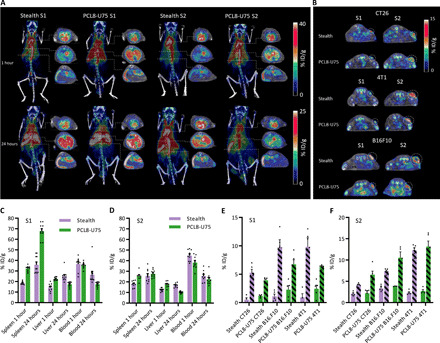
PET/CT imaging of radiolabeled PCL8-U75 ^64^Cu-liposomes and stealth ^64^Cu-liposomes. (**A**) Coronal maximum intensity projections and axial slices at the level of the heart (top), liver (middle), and spleen (bottom) as illustrated by dashed lines of PET/CT scans performed 1 and 24 hours after first (S1) and third (S2) injection of stealth L-OHP liposomes or PCL8-U75 L-OHP liposomes including radiolabeled liposomes (*n* = 5). (**B**) Axial PET/CT images illustrating PCL8-U75 ^64^Cu-liposome and stealth ^64^Cu-liposome activity in CT26, 4T1, and B16.F10 tumors on the 24-hour images of S1 and S2 (dashed circles). (**C** and **D**) Biodistribution of PCL8-U75 and stealth ^64^Cu-liposomes at S1 (C) and S2 (D) in spleen, liver, and blood 1 and 24 hours after injection. The data represent activity in Balb/cJRj and C57BL/6JRj mice independent of tumor model. (**E** and **F**) Tumor accumulation 1 hour (plain) and 24 hours (dashed) after injection of PCL8-U75 and stealth ^64^Cu-liposomes at S1 (E) and S2 (F) in CT26, 4T1, and B16.F10 tumors.

The PCL8-U75 liposomes must have a long circulating half-life to achieve the EPR-mediated accumulation in solid tumors (*27*). To investigate this, we performed positron emission tomography (PET)/computed tomography (CT) imaging using radiolabeled (^64^Cu; *T*_1/2_ = 12.7 hours) liposomes (*28*). A mix of radiolabeled and L-OHP–loaded liposomes [L-OHP (8 mg/kg)] of PCL8-U75 or stealth liposomes was injected to provide theranostic data on tumor accumulation when using therapeutic doses of L-OHP. Circulation and biodistribution of PCL8-U75 liposomes were evaluated in Balb/cJRj mice bearing CT26 or 4T1 tumors and in C57BL/6JRj mice bearing B16.F10 tumors (Fig. 2, A and B). On the basis of PET imaging, it was identified that the shielding of the cationic lipids by PCL was sufficient to achieve long circulating (Fig. 2A) and tumor-accumulating properties when evaluated by PET/CT imaging (Fig. 2, B, E, and F). Furthermore, it is central for liposomes to maintain their circulating and tumor-accumulating properties throughout the course of therapy. To investigate how the circulating properties of PCL8-U75 L-OHP and stealth L-OHP liposomes were affected by repeated dosing, we also performed the PET/CT scanning procedure with mixed radiolabeled and L-OHP liposomes in connection with the third therapeutic liposome administration (Fig. 2A). Here, the circulation properties were maintained for both stealth and PLC8-U75 liposomes, indicating that no immunological reactivity toward either liposome formulation was raised during the interscan period. The biodistribution of the radiolabeled PCL8-U75 liposomes was comparable to the stealth liposomes with the exemption of higher activity in the spleen, especially at the first injection (Fig. 2, C and D). EPR-dependent tumor accumulation was evident for both liposome formulations in all three syngeneic cancer models, i.e., tumor activity increased between the 1- and 24-hour time points, which shows that EPR-mediated liposome accumulation occurs (Fig. 2B). At the first administration, a tendency toward higher tumor accumulation of radiolabeled stealth liposomes compared to PCL8-U75 liposomes was observed at the 24-hour PET scan, whereas the opposite trend was observed after the third liposome injection (Fig. 2, E and F). In general, there was slightly higher tumor activity at the third administration of liposomes, which could be associated with either longer circulation time or increased liposome extravasation into tumors following chemotherapy exposure. ^64^Cu-liposomes had a high level of accumulation in all three syngeneic cancer models.

### Potent antitumor activity in syngeneic cancer models

**Fig. 3:**
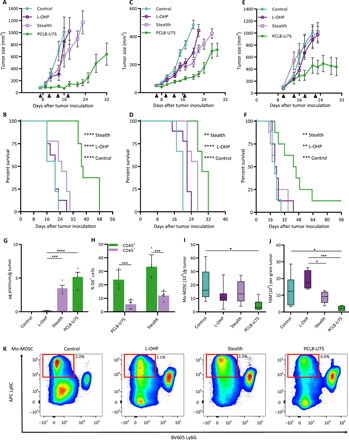
Effect of L-OHP PCL8-U75 in syngeneic tumor models with high tumor accumulation in leukocytes and depletion of immunosuppressive populations. Three syngeneic tumor models were treated with L-OHP (8 mg/kg), PCL8-U75 L-OHP, and stealth L-OHP liposomes every 4 days as indicated by arrowheads. Tumor growth curves are shown as mean tumor size ± SEM. Tumor growth curve and Kaplan-Meier plots of B16.F10 tumors on C57BL/6JRj (**A** and **B**), orthotopic inoculated 4T1 tumors on Balb/cJRj mice (**C** and **D**), and CT26 tumors on Balb/cJRj mice (**E** and **F**). Median survival time of PCL8-U75 L-OHP–treated groups was significantly improved compared to L-OHP stealth, free L-OHP, and untreated controls across all tumor models (**P < 0.005 and ****P < 0.0001). *n* = 8 to 10. (**G**) Tumor levels of platinum were investigated 48 hours after the second treatment with L-OHP PCL8-U75, stealth L-OHP, or free L-OHP. The platinum levels were significantly higher for both stealth- and PCL8-U75 liposome–treated tumors (***P < 0.001 and ****P < 0.0001). *n* = 3 to 5. (**H**) Bar plot showing the association of CD45^+^ and CD45^−^ populations and the DiI signal. Both liposome formulations were stably formulated with DiI and L-OHP, and the association in immune cells was analyzed 24 hours after intravenous injection of L-OHP (8 mg/kg). The percentage of DiI^+^ cells from the leukocytic CD45^+^ or cancer cell CD45^−^ (fsc^mid^ ssc^mid^) populations is shown as means and SEM (***P < 0.001). *n* = 5. CT26 TME analysis by flow cytometry and the count per gram of tumor of Mo-MDSCs (**I**) and TAMs (**J**) was performed 48 hours after the third treatment with L-OHP PCL8-U75, L-OHP stealth, L-OHP, or untreated controls. (I) Mo-MDSCs (CD11b^+^ CD11c^−^ Ly6C^hi^ Ly6G^lo^) cells were significantly lower in the PCL8-U75 L-OHP group (*P* < 0.05). *n* = 6 to 10. (J) PCL8-U75 depletes TAM in the TME compared to free L-OHP and stealth L-OHP (*P < 0.05 and ***P < 0.001). *n* = 6, mean and SEM are shown. (**K**) Representative pseudocolor plots from CT26 tumor samples. Parent gate is the CD45^+^ CD11b^+^ CD11c^−^ population, and the red gate shows the Ly6C^hi^-defined Mo-MDSCs. The number indicated is the percent of scatter gate.

On the basis of the intricate association between ICD-inducing chemotherapeutics and immune activation, the therapeutic efficacy of the PCL liposomes was investigated in syngeneic cancer models in immunocompetent mice. Free drug or liposomal L-OHP was injected intravenously (8 mg/kg) at 4-day intervals for four treatments. PCL8-U75 L-OHP liposomes displayed impressive therapeutic efficacy in large established subcutaneous CT26, B16.F10, and orthotopic 4T1 tumors compared to L-OHP and stealth L-OHP liposomes (Fig. 3, A to F, and figs. S4 to S6). The groups treated with PCL8-U75 L-OHP liposomes displayed improved tumor control compared to both free drug and stealth L-OHP liposomes, which was evident following the second treatment across all three models evaluated. In addition, the L-OHP PCL8-U75 group had significantly increased median survival time compared to the L-OHP stealth group in all tumor models (CT26, *P* = 0.004; B16.F10, *P* = 0.00005; 4T1, *P* = 0.001; Fig. 3, B, D, and F) and no weight loss following PCL8-U75 treatment (fig. S7, A to C). One mouse in the PCL8-U75 L-OHP showed complete rejection of the tumor and was able to reject a cancer cell rechallenge >100 days after initial inoculation (fig. S8C).

To decipher these results, we further analyzed the TME. On the basis of the hypothesized interaction between cationic liposomes and myeloid cells, mechanistic studies were conducted in the CT26 model as the TME in this model is dominated by Mo-MDSCs and suppressive TAM populations (*29*). Initially, we substantiated our PET-based biodistribution results by measuring the tumor platin level 48 hours after the second administration of free L-OHP, stealth L-OHP liposomes, or PCL8-U75 L-OHP liposomes. The drug delivery potential of the L-OHP liposome formulations was apparent by the high tumor platin levels compared to free L-OHP (Fig. 3G). There was no significant difference in platin levels between tumors treated with stealth L-OHP liposomes or PCL8-U75 L-OHP liposomes, evidencing that improved bioavailability and potential immune activation are the mechanisms behind the superior antitumor activity and not on improved tumor accumulation. To track liposomal content in the TME, we labeled PCL8-U75 and stealth liposomes with a lipidated fluorophore [1,1′-dioctadecyl-3,3,3′,3′-tetramethylindocarbocyanine perchlorate (DiI)]. Single-cell analysis from the tumors was performed 24 hours after first injection. Both PCL8-U75 and stealth liposomes accumulated primarily in CD45^+^ leukocytes (Fig. 3H and fig. S8A). However, the depletion potential of suppressive CD11b^+^ CD11c^−^ Ly6c^hi^ Ly6G^low^ Mo-MDSC and CD11b^+^ CD11c^+^ CD64^hi^ TAMs was distinctively different between the two-liposome formulations (Fig. 3, I and J). This observation is highly important as it shows that the drug availability and activity of PCL8-U75 L-OHP liposomes are distinctly different to that of stealth liposomes, supporting that the PCL formulation has potent anticancer and immune modulating properties.

The free L-OHP–treated group showed a tendency of low levels of Mo-MDSC in the TME with large variation (Fig. 3, I and K), which could be an effect of the systemic myelotoxicity, commonly observed for L-OHP (*30*). Whole blood analysis following third treatment confirmed that free L-OHP depletes circulating inflammatory monocytes (CD11b^+^ Ly6C^hi^) (fig. S8B) that are part of the progenitor pool of tumor-infiltrating myeloid cells.

This is different for the PCL8-U75 formulation, which does not eliminate myeloid cell linages in circulation, but depletes the TME of both Mo-MDSC and TAMs (Fig. 3, I to K, and fig. S8B). The ability of PCL8-U75 to deplete suppressive populations in the TME without concurrent bone marrow suppression is critical to the toxicity profile and the potency as an immunotherapeutic adjuvant.

#### Synergistic effects with immune-activating TLR7

**Fig. 4:**
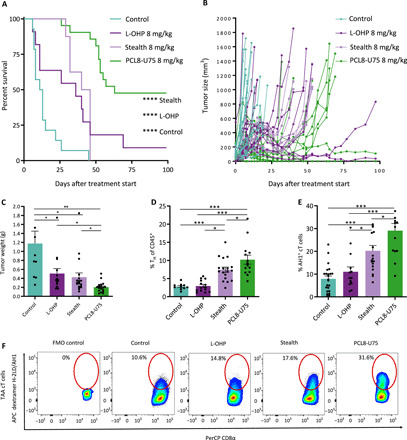
Synergistic effects of L-OHP PCL8-U75 and R848 induce pro-immunogenic infiltration and rejection of CT26 tumors. Mice carrying CT26 tumors received three injections with free L-OHP, PCL8-U75 L-OHP, or stealth L-OHP liposomes (8 mg/kg) every 3 days followed by four intravenous injections of R848 (3 mg/kg) at 3-day intervals. The Kaplan-Meier plot (**A**) and individual tumor growth curves (**B**) demonstrate increased survival of the PCL8-U75 L-OHP group in comparison to the L-OHP and stealth L-OHP and control groups. The plots contain data from three independent experiments. Complete responders (CR) and median survival time (MST): control: CR, 0 of 14; MST, 12 days; free L-OHP: CR, 1 of 11; MST, 36 days; stealth: CR, 0 of 8; MST, 43 days; PCL8-U75: CR, 10 of 22; MST, 63 days (****P < 0.0001). CT26 tumors from mice receiving combined treatment were analyzed by flow cytometry, and data are shown in (C) to (F). Control tumors (*n* = 10), L-OHP (*n* = 14), stealth L-OHP (*n* = 19), and PCL8-U75 L-OHP (*n* = 17). (**C**) Tumor weights at the time of analysis. Significantly lower tumor mass in the L-OHP R848–treated groups compared to controls and the PCL8-U75 L-OHP R848 were significantly smaller than both the L-OHP R848– and stealth L-OHP R848–treated group (**P < 0.01 and *P < 0.05). (**D**) Flow cytometry analysis of T helper (T_H_) cells CD45^+^ CD3^+^ CD4^+^ FoxP3^−^. PCL8-U75 L-OHP + R848 had significantly higher infiltration compared to stealth L-OHP–treated tumors (***P < 0.001 and *P < 0.05). (**E**) Tumor-associated antigen-specific cT cells CD45^+^ CD3^+^ CD8^+^ dexH-2Ld/AH1^+^ were significantly increased for the liposomal L-OHP + R848 groups in comparison to the L-OHP + R848 and control groups. The PCL8-U75 L-OHP R848 combination was superior to the stealth L-OHP R848 in establishing a tumor antigen–specific response (***P < 0.001 and *P < 0.05). (**F**) Analysis of tumor-specific cT shown by AH1 dextramer^+^ (H-2LD/AH1) cT cells. Fluorescence minus one (FMO) control of H-2LD/AH1 APC is included.

The anticancer and immune-modulating properties of the PCL8-U75 L-OHP liposomes are attractive for combinations with immune-activating therapies. The cytotoxic mechanism of L-OHP is expected to provide tumor antigens and subsequent ICD-mediated immune activation. This should, in combination with the potent depletion of immunosuppressive Mo-MDSC and TAMs by PCL8-U75 L-OHP liposomes, result in optimal synergy with immunostimulatory agents. To investigate this, we combined the PCL8-U75 L-OHP liposomes with the TLR7 agonist R848, which has previously shown synergistic effects with ICD-inducing radiation therapy (*31*, *32*). Treatment with free L-OHP, stealth L-OHP, or PCL8-U75 L-OHP liposomes (8 mg/kg; three injections, 3-day interval) was combined with subsequent administrations of free R848 intravenously (3 mg/kg) at 3-day intervals for four treatments. The PCL8-U75 L-OHP liposomes showed potent synergistic effects compared to both stealth L-OHP liposomes and free L-OHP in combination with R848. The addition of R848 had clear effect on the observed responses in the PCL8-U75 L-OHP liposome group as 45% (10 of 22) of the mice had complete responses compared to none in the stealth L-OHP liposome (0 of 8) and 9% (1 of 11) in the free L-OHP group (Fig. 4, A and B). All complete responders displayed immunologic rejection of a cancer cell rechallenge 100 days after the first inoculation with naïve mice included to verify cell tumorigenicity (fig. S7C). This demonstrates that the therapeutic intervention has induced immunological memory to vaccinate mice against identical cancer cells. Together, the results clearly demonstrate that the L-OHP in the PCL8-U75 liposomes provides improved ICD with subsequent immune activation with the ability to deplete suppressive protumorigenic myeloid populations. To investigate the development of an adaptive anticancer response, we analyzed the TME 4 days after completing L-OHP and R848 treatments. The superior therapeutic efficacy of the PCL8-U75 L-OHP liposomes was evident by the significantly lower mean tumor weight in comparison to all other treatments (Fig. 4C). The tumor-associated T helper (T_H_) cells (CD3^+^ CD4^+^ FoxP3^−^) are central in augmenting an optimal adaptive anticancer response and a significant increase in PCL8-U75 L-OHP liposome–treated tumors compared to all other treatments (Fig. 4D). Infiltration of cT cells with H-2LD specificity against the immunodominant epitope AH-1 (CD3^+^ CD8^+^ AH1^+^) (*33*) in CT26 tumors is central to the anticancer response. Encouragingly, the PCL8-U75 L-OHP–treated tumors had significantly higher infiltration of this population compared to all other groups, which directly identifies the potential of the PCL8-U75 L-OHP system as part of an immune activation therapeutic protocol (Fig. 4, E and F).

## DISCUSSION

Low bioavailability of encapsulated drugs, particularly platinum-based drugs, is a well-recognized Achilles heel for liposomal drug delivery systems, which hampers the possibilities to exploit the potential of the high accumulation levels in solid cancers. The PLC8-U75 tumor-targeting liposomes are attractive treatments as they provide increased levels of cancer antigens and ICD-mediated immune activation compared to the free drug. Stimulation of a sufficient antitumoral immune response is, however, a continuous battle between the ICD-induced immune activation and the intratumoral immunosuppression. We therefore aimed to design a liposomal L-OHP formulation that provides extensive and coordinated drug release to achieve improved cytotoxicity and immune-activating properties. Liposomal platinum-based formulations have been tested extensively in preclinical settings (*34*, *35*), but only few have reached clinical testing (*19*, *36*, *37*). In the case of cisplatin, the liposomes appeared to be too stable, and as a consequence, they suffered from poor drug release (*38*) or were associated with increased toxicity without indications of improved therapeutic effect (*39*). Considering the passive EPR-mediated liposome accumulation in tumors, excellent therapeutic efficacy should be achievable with optimized liposomal drug release upon tumor accumulation. At the intracellular level, PEGylation has been found to interfere with endosomal escape and trafficking (*20*), which potentially limits the therapeutic potential of a PEGylated drug carrier. We constructed the presented PCL8-U75 L-OHP liposomes to overcome the problems associated with liposomal chemotherapeutic drug delivery systems. The PCL8-U75 successfully achieved optimal systemic circulation and tumor-accumulating properties even at repeated administrations while having potent in vitro and in vivo therapeutic efficacy. There was no difference in the accumulation of PCL8-U75 in CD45^+^ cells compared to stealth, indicating that increase of L-OHP bioavailability from PCL8-U75 is more complex than just increased cell interaction (Fig. 3H).

The association between immune cell infiltration and prognosis for clinical cancer patients is providing extensive insights into the central importance of the TME (*40*). In this respect, the observed intratumoral depletion of immunosuppressive TAMs and Mo-MDSC by the PCL8-U75 L-OHP liposomes is highly encouraging. The PCL8-U75 L-OHP liposomes were able to deplete both of these suppressive subsets in CT26 tumors without lowering the circulatory level of monocytes (Fig. 3, I and J, and fig. S7B). This is in contrast to free L-OHP treatment with low bioavailability due to low tumor accumulation and the stealth liposome formulation with too high L-OHP stability (*36*).

The observed efficacy of PCL8-U75 L-OHP in combination with a TLR7 agonist (R848) indicate that the immune-modulating properties provide a substantial advantage from a therapeutic point of view. R848 polarizes myeloid populations toward a pro-immunogenic phenotype with improved antigen-presenting ability and increased release of pro-inflammatory cytokines (*41*). The depletion of suppressive myeloid populations by the PCL8-U75 L-OHP liposomes allows infiltration of more pro-immunogenic population subtypes following TLR7 stimulation. The transition to an immune-supportive TME led to increase of T_H_ and AH1 tumor-specific cT cells in the PCL8-U75 L-OHP liposome–treated group compared to all other treatments (Fig. 4, D and E). AH1 is the immunodominant cT cell epitope and stems from overexpressed gp70 in CT26 cells (*42*). This illustrates that not only are cancer cells killed by cytotoxic effects of the delivered L-OHP, but also the therapeutic combination with R848 improves the generation of tumor-specific T cells and supports their tumor infiltration and activity. The strategy to deplete suppressive myeloid populations and induce ICD of cancer cells with an optimized drug delivery and release system holds promise for their introduction in future immunotherapeutic combinations. The combination of L-OHP and R848 led to weight loss (<10%) compared to untreated controls, which is a well-established effect observed in connection with systemic R848 therapy (*31*). There was no difference in weight between the PCL8-U75, stealth, or free L-OHP (fig. S7, A to D).

The encouraging data presented here point to a new avenue for liposome drug delivery systems, where impressive immune-modulating properties may provide optimal basis for boosting the anticancer immune response, even with conventional ICD-inducing chemotherapeutics. Additional studies aiming to identify the optimal immune-stimulating and immune-modulating effects of advanced liposomal drug delivery systems are therefore highly warranted and have high potential for clinical translation.

## MATERIALS AND METHODS

POPC; DSPC; DOTAP; DSTAP; cholesterol; and 1,2-distearoyl-*sn*-glycero-3-phosphoethanolamine-*N*-[amino(PEG)-2000] (DSPE-PEG2000) were purchased from Avanti Polar Lipids Inc. (Alabaster, AL, USA). ^3^H-cholesterylhexadecyl ether (^3^H-CHE) was from PerkinElmer (Skovlunde, Denmark). 3-(4,5-Dimethylthiazol-2-yl)-5-(3-carboxymethoxyphenyl)-2-(4-sulfophenyl)-2H-tetrazolium (MTS) was purchased from Promega. DQ gelatin was from Life Technologies (Taastrup, Denmark). 9-Fluorenylmethoxycarbonyl (Fmoc) amino acids and *O*-(7-azabenzotriazol-1-yl)-1,1,3,3-tetramethyluronium hexafluorophosphate (HATU) were purchased from GL Biochem (Shanghai, China) or Bachem AG (Bubendorf, Switzerland). TentaGel PAP_2000_ resin was purchased from Rapp Polymere GmbH (Tuebingen, Germany). DiI was purchased from Thermo Fisher Scientific. Oxaliplatin (L-OHP) was from Lianyungang Guiyuan Chempharm Co. Ltd. (Jiangsu, China). 1,4,7,10-Tetraazacyclododecane-1,4,7,10-tetraacetic acid (DOTA) was purchased from Macrocyclics (Dallas, TX, USA). All other chemicals were from Sigma-Aldrich (Brøndby, Denmark) and of analytical grade.

### Synthesis, purification, and characterization of the PCL

The peptide H-Gly-Trp(Boc)-Ile-Pro-Val-Ser(*t*Bu)-Leu-Arg(Pbf)-Ser(*t*Bu)-Gly-Glu(*t*Bu)-Glu(*t*Bu)-Glu(*t*Bu)-Glu(*t*Bu) was synthesized on an Initiator Alstra peptide synthesizer (Biotage, Uppsala, Sweden) using a TentaGel PAP_2000_ resin. The resin was swelled in dichloromethane (DCM) for 1 hour. In general, couplings were 5 min at 75°C using 4 eq. of amino acid, 3.92 eq. of HATU, and 8 eq. of 2,4,6-collidine in *N*,*N*′-dimethylformamide (DMF). Ile was double coupled. The second coupling was 30 min at room temperature. Arg was coupled 30 min at room temperature. Fmoc deprotection was done using 20% piperidine in DMF for 3 plus 10 min. The peptide was cleaved for 3 hours using 95:2.5:2.5 trifluoroacetic acid (TFA)/water/triisopropyl silane after which the cleavage solvent was removed under reduced pressure, and the peptide precipitated in diethyl ether. The cleaved peptide was dissolved in 5% dimethyl sulfoxide in water and purified using semipreparative high-performance liquid chromatography (HPLC) (Waters 600 Pump and Controller and a Waters 2489 UV/Visible Detector) using a Waters XTerra C_18_ 5-μm (19 mm × 150 mm) column. Eluent: (A) 5% acetonitrile, 0.1% triethylamine (TEA) in water, and (B) 0.1% TEA in acetonitrile. Gradient profile: linear gradient from 0% B to 50% B over 15 min. Flow rate: 17 ml/min. The PEGylated peptide was isolated as a broad peak with retention time around 8 min. The product was lyophilized to give a white powder. The peptide was dissolved in dry NMP (N-Methyl-2-pyrrolidone) in 1 mM concentration. To the dissolved peptide was added 1.2 eq. of cholesteryl chloroformate and 20 eq. of diisopropylethylamine, and the solution was stirred. After 30 min, the reaction was diluted to 10% NMP with water and purified using semipreparative HPLC as described above. PCL was isolated as a broad peak with retention time around 12 min. The product was lyophilized to give a white powder. The purity of the product was monitored by analytical HPLC using a Waters XTerra C_8_ 5-μm (4.6 mm × 150 mm) column. Eluent: (A) 5% acetonitrile, 0.1% TFA in water, (B) 0.1% TFA in acetonitrile. Gradient profile: linear gradient from 0% B to 100% B over 15 min. Flow rate: 1 ml/min. Purity: >95%. Rt (retention time): 12.3 min.

### Preparation of liposomes

Liposomes were prepared by dissolving lipids in *tert*-butanol:water (9:1) followed by freeze-drying. The following molar ratios were used: DSPC:cholesterol:DSTAP:PCL: 55:32:5:8 (PCL8-S50), 52.5:32:7.5:8 (PCL8-S75), POPC:cholesterol:DOTAP:PCL: 55:32:5:8 (PCL8-U50), and 52.5:32:7.5:8 (PCL8-U75). Stealth liposomes consisted of HSPC:cholesterol:DSPE-PEG2000 at the molar ratio of 55:40:5.

The dry lipid powder was hydrated in a saturated L-OHP solution [15 mg/liter in 10 mM Hepes and 5% glucose (pH 7.4)] for 1 hour at 65°C. The resulting liposomes were extruded with a high-pressure extruder (Northern Lipids Inc., Burnaby, Canada) by passing the liposomes two times through two stacked 200-nm polycarbonate filters (Whatman, Maidstone, UK) followed by five passes through two 100-nm filters. Alternatively, for smaller preparations, the lipid suspensions were extruded 21 times through a 100-nm polycarbonate filter using a mini-extruder (Avanti Polar Lipids, Alabaster, AL, USA) to form unilamellar liposomes. The temperature was maintained at 65°C during the extrusion process. Unencapsulated free L-OHP was removed by dialysis using a dialysis cassette (Slide-A-Lyzer, 10,000 molecular weight cutoff (MWCO), Pierce, Thermo Fisher Scientific, Slangerup, Denmark) against 10 mM Hepes and 5% glucose (pH 7.4). Encapsulated L-OHP and phospholipid content were measured by inductively coupled plasma mass spectrometry (ICP-MS) (ICAPq, Thermo Fisher Scientific, Hvidovre, Denmark) using Iridium or Gallium as internal standards. The PCL construct was inserted to the outer liposomal membrane using post-insertion technique. Here, liposomes were added to freeze-dried PCL construct and subjected to gentle stirring for 45 min at 40°C. To measure the degree of encapsulation, the liposomes were subjected to spin-filtration (Amicon Ultra, 100 K, Merck Life Science, Denmark), and the L-OHP concentration was measured in the filtrate. The particle size and charge were determined with a Zetasizer (Brookhaven Instruments Ltd., NY, USA). Digestion with protease, 20 μl of liposomes was mixed with 180 μl of Hepes-buffered saline [100 mM NaCl, 50 mM Hepes (pH 7.4), 1 mM CaCl_2_, and 2 μM ZnCl_2_ supplemented with thermolysin (20 μg/ml) (stable surrogate for MMP9 with same peptide cleavage site)]. Enzymatic cleavage was performed overnight at 37°C.

### Loading of ^64^Cu into liposomes

Liposomes entrapping the high-affinity copper chelator, DOTA, were prepared. Briefly, freeze-dried lipid powder (50 mg/ml; stealth or PCL8-UU75) was dispersed in a buffer containing 10 mM DOTA, 10 mM Hepes, and 150 mM NaCl (pH 7.4). The lipid suspension was hydrated for 60 min at 65°C and subsequently sized to 100 nm using a mini-extruder (Avanti Polar Lipids). The nonencapsulated DOTA was removed by dialysis [Slide-A-Lyzer, 10,000 MWCO, Pierce, Thermo Fisher Scientific, Slangerup, Denmark] against a buffer containing 10 mM Hepes and 150 mM NaCl (pH 7.4). For remote loading, liposomes entrapping DOTA were added to a vial containing dried ^64^CuCl_2_. The liposome sample was incubated for 75 min at 65°C using constant stirring and following equilibrated at room temperature. The ^64^Cu loading efficiency was >95%, determined by radio-HPLC and radio–thin-layer chromatography (*43*). Before administration, the ^64^Cu-loaded liposomes were diluted sixfold with either empty or L-OHP–loaded liposomes reaching a final activity concentration of 50 MBq/ml corresponding to 10 MBq per mouse.

### Cryo-TEM imaging

Electron microscopy studies were performed using a CM120 BioTWIN transmission electron microscope (Philips, Amsterdam, The Netherlands), with a cryo-holder and a cryo-transfer stage as described in (*44*). Briefly, a thin film of sample solution was prepared by a blotting procedure, performed in a custom-built environmental chamber with controlled temperature (25°C) and humidity. A drop of the solution was placed onto a lacy carbon film supported by a copper grid. The copper grid was blotted with filter paper leaving a thin film of the sample solution to span the holes of the carbon grid. The thin film was rapidly frozen by plunging the grid into liquid ethane (−180°C). The frozen specimen was subsequently transferred to the microscope. The temperature was kept below −165°C during both the transfer and viewing procedures to prevent sample perturbation and formation of ice crystals. Samples tested had a lipid concentration of 10 mM.

### Cell culture and in vitro toxicity

The cancer cell lines, CT26 (murine colon carcinoma), B16F10 (murine melanoma), FaDu [Human head and neck (H&N)], and 4T1 (murine mammary carcinoma), were all purchased from American type culture collection (ATCC) (Rockville, MD, USA) and cultured in RPMI or Dulbecco’s modified Eagle’s medium supplemented with 10% fetal calf serum and 1% penicillin-streptomycin (Invitrogen Inc., Denmark) at 37°C and 5% CO_2_ in a humidified atmosphere. Cell lines were kept <10 passages from thawing and tested for STR (short tandem repeat) profile and interspecies contamination in December 2018. Mycoplasma testing is performed annually. Sensitivity to the L-OHP formulations was evaluated using MTS assay. Briefly, cells were seeded onto 96-well plates at a density of 4000 cells per well the day before drug treatment. The culture medium was replaced by medium containing increasing amounts of the indicated drugs. After 72-hour incubation, the medium was removed, and the cells were incubated with MTS solution until sufficient amount of color was developed and within the linear range. The absorbance was read at 490 nm using a microplate reader (Sunrise, Tecan, USA).

### PET scanning using ^64^Cu-loaded liposomes to study biodistribution

Female Balb/cJRj or C57BL/6JRj mice (Janvier Laboratories, Le Genest-Saint-Isle, France), 7 to 12 weeks old, were inoculated subcutaneously on the flank with 3 × 10^5^ cells in 100 μl of RPMI. Tumors were allowed to reach a size of approximately 150 mm^3^ before being randomized. Mice were divided into four treatment groups, and half the mice from each group were scanned with either the ^64^Cu-loaded stealth liposome formulation or the ^64^Cu-loaded PCL8-U75 liposome formulation. The mice received four doses of drug or vehicle at 4-day intervals. Combined injection of L-OHP treatment formulation and ^64^Cu-loaded liposomes was performed for treatment 1 and 3.

For the imaging procedures, mice received intravenous injection of treatment (vehicle, L-OHP, stealth, or PCL8-U75) and ^64^Cu-loaded liposomes intravenously, and combined PET/CT imaging was performed after a distribution period of 1 hour (termed 1-hour scan) and 24 hours (termed 24-hour scan). Mice received a mean dose of ^64^Cu-loaded liposome of 9.9 MBq (SD, 1.6 MBq; SEM, 0.19 MBq), and all mice received a lipid dose of 3.3 mM from the radiolabeled liposome formulations. Mice were allowed to cage rest during the distribution periods. PET/CT imaging was performed on a dedicated small-animal PET/CT system with CT-based PET image attenuation (Inveon, Siemens Medical Systems, Malvern, PA, USA). Mice were anesthetized using 3.5% sevoflurane in 40% O_2_ in medical grade air and placed on heating pads to secure body temperature during the imaging procedures. PET/CT scans were performed as 5-min (1-hour scan) and 15-min (24-hour scan) PET scans followed by a CT scan (tube setting, 70 kV and 500 μA; exposure time, 350 ms; pixel size, 0.21 mm by 0.21 mm by 0.21 mm). PET data were arranged into sinograms and reconstructed using a maximum a posteriori reconstruction algorithm (pixel size, 0.388 mm by 0.388 mm by 0.796 mm) with attenuation correction based on the corresponding CT scan.

Image analysis was performed using Inveon software (Siemens Medical Systems, Malvern, PA, USA). Regions of interest (ROIs) were manually constructed on the basis of the co-registered PET/CT images. The following ROIs were constructed: tumors (complete volume delineated), liver, spleen, muscle, urinary bladder lumen, kidney, lung, and blood. Blood activity was estimated from a constructed volume of interest ROI covering the left ventricular lumen of the heart, which were subsequently segmented to only include the voxels displaying above 80% of maximum activity with the original ROI. ROIs were constructed by multiple axial gating to avoid dense vascularized irrelevant regions in order to remove false positive signal from blood vessels.

### Animal experiments: Tumor accumulation of L-OHP

To evaluate tumor accumulation of L-OHP after liposome or free-drug administration, Balb/cJRj mice carrying CT26 tumors or NMRI nude mice (Taconic, Denmark) carrying FaDu tumors (5 × 10^6^ cells in 100 μL of RPMI media inoculated subcutaneously on the flank) were injected intravenously with 8 mg of L-OHP/kg. Liposomes containing encapsulated L-OHP were injected intravenously (8 mg L-OHP/kg). After the mice were sacrificed, tumors were dissected and snap-frozen. To measure platinum content in tumors, 50 to 100 mg of tissue was added to 15-ml Falcon tube and weighed before being digested overnight at 65°C in 500 μl of HNO_3_, 50 μl of HCl, and 300 μl of H_2_O_2_. Ten milliliters of MQ (mill-q) water was added, and after weighing, the samples were further diluted in 2% HCl. The platinum content in the tissues was measured by ICP-MS.

### Animal experiments: Efficacy and toxicity

All experimental procedures were approved by the institutional ethical board 141 and the Danish National Animal Experiment Inspectorate. CT26 cancer cells (3 × 10^5^) were injected (100 μL, in RPMI media) subcutanously on the flank of Balb/cJRj mice, 4T1 cancer cells (1 × 10^5^) were injected (50 μL, in RPMI media) in the mammary fatpad of Balb/cJRj mice, and B16F10 cancer cells (2 × 10^5^) were injected (100 μL in RPMI media) subcutaneously in the flank of C57BL/6JRj mice. When tumors reached mean of 100 mm^3^, the animals were randomized and blocked for size. After randomization, the mice received four intravenous administrations every 4 days. The mice were weighed three times a week during the study as a measurement of toxicity. The tumor volumes were measured three times a week using an electronic caliper. Tumor volume was calculated according to the formula: (*a* × *b*^2^)/2 (*a* = length, *b* = width). The animals were sacrificed when the tumor volumes reached 1500 mm^3^ for flank tumors or 500 mm^3^ for orthotopic tumors. Humane endpoints included signs of misthriving animals or excessive weight loss (>15% of start weight or >10% overnight weight loss). No animals were sacrificed because of toxicity-related symptoms.

The study has been approved by The Danish Animal Experiments Inspectorate.

### Flow cytometry

Antibodies to CD3ε (1-7A2), CD4 (RM4-5), CD8α (53-3.7), CD25 (PC61.5), CD45 (30-F11), CD11c (N418), and FoxP3 (FJK-16 s) were purchased from eBioscience. Ly-6G (1A8) was purchased from BioLegend. Ly-6C (AL-21) and CD11b (M1/70) were purchased from BD Biosciences. AH1 dextramer (H-2 Ld SPSYVYHQF) was bought from IMMUDEX. eBioscience Fixable Viability Dye eFluor 780 was used to assess viability. Foxp3 was stained using the Foxp3/transcription factor staining buffer set from eBioscience. Analysis following monotherapy of L-OHP was performed 48 hours after third treatment. Analysis following combination treatment was done at day 27 after inoculation for L-OHP and untreated tumors and day 29 for PCL8-U75 liposomes– and stealth-treated groups. Tumors were resected and stored in MACS tissue storage solution (Miltenyi Biotec) on ice. Subsequently, they were weighed and placed in enzymatic murine tumor dissociation solution (Miltenyi Biotec). All tumors below 90 mg were pooled. Next, samples were dissociated by shaking for 40 min at 37°C in a water bath with agitation. Following enzymatic digestion, the tumors were passed through 70-μm cell strainers twice to achieve single-cell suspensions. The remaining cells were diluted in phosphate-buffered saline, and the cell concentration of the samples was determined using MUSE Cell Count and Viability Assay on a MUSE Cell Analyzer (Merck Millipore) according to the manufacturer’s guidelines. For flow analysis, 10 to 20 × 10^6^ cells were Fc-blocked (5 min on ice) with murine anti-CD16/32 monoclonal antibodies, followed by labeling of dead cells with fixable viability dye–efluor 780 and surface staining (30 min at 4°C) with specific antibodies. Samples were run on either BD FACSCanto II or LSR Fortessa X-20 by acquiring 1 × 10^6^ to 2.5 × 10^6^ events. Data analysis was performed with FlowJo 10.0 and GraphPad Prism 8.0. To compensate for spectral spillover, compensation controls were included using the AbC Total Antibody Compensation Bead Kit (Life Technologies, Thermo Fisher Scientific). Fluorescence minus one (FMO) samples were included and used for correct identification of positive and negative populations. Gating strategy is shown in fig. S10 (A to C).

#### *Analysis of DiI liposome uptake*

Balb/cJRj mice were inoculated subcutaneously on the right flank with 3 × 10^5^ CT26 cells in 100 μl of RPMI. The mice received DiI (0.25 mole percent)–labeled liposomes at 230 μmol of lipid/kg, corresponding to oxaliplatin (8 mg/kg) in PCL8-U75 or stealth formulation intravenously, when tumors reached a mean of 200 mm^3^. Flow analysis was performed 24 hours after administration using the method described above. Untreated mice were used as FMO controls to set the positive gates.

#### *Statistical analysis*

Statistical analysis was performed in Prism 8 (GraphPad Software) except for flow data, which were first gated using FlowJo and subsequently analyzed in Prism. Survival was analyzed by the log-rank (Mantel-Cox) test. Comparison between groups was performed with one-way analysis of variance (ANOVA) with Tukey post hoc test for multiple comparisons. All data are presented as the means ± SEM, unless otherwise stated. A *P* value <0.05 was considered statistically significant.

## Supplementary Material

aba5628_SM.pdf

## SUPPLEMENTARY MATERIALS

Supplementary material for this article is available at http://advances.sciencemag.org/cgi/content/full/6/36/eaba5628/DC1

## Appendix

View/request a protocol for this paper from *Bio-protocol*.
