# Supplementary material for: Tumor repolarization by an advanced liposomal drug delivery system provides a potent new approach for chemo-immunotherapy
Source: Sci Adv. 2020 Sep 4;6(36):eaba5628. doi: 10.1126/sciadv.aba5628 (PMC7473747; doi:10.1126/sciadv.aba5628)
Supplement: aba5628_SM.pdf [file aba5628_SM.pdf]

Science Advances
AAAS

advances.sciencemag.org/cgi/content/full/6/36/eaba5628/DC1

Supplementary Materials for

Tumor repolarization by an advanced liposomal drug delivery system provides a potent new approach for chemo-immunotherapy

L. Ringgaard, F. Melander, R. Eliasen, J. R. Henriksen, R. I. Jølck, T. B. Engel, M. Bak, F. P. Fliedner, K. Kristensen, D. R. Elema, A. Kjaer, A. E. Hansen, T. L. Andresen*

*Corresponding author. Email: tlan@dtu.dk

Published 4 September 2020, *Sci. Adv.* **6**, eaba5628 (2020)
DOI: 10.1126/sciadv.aba5628

This PDF file includes:

Figs. S1 to S10

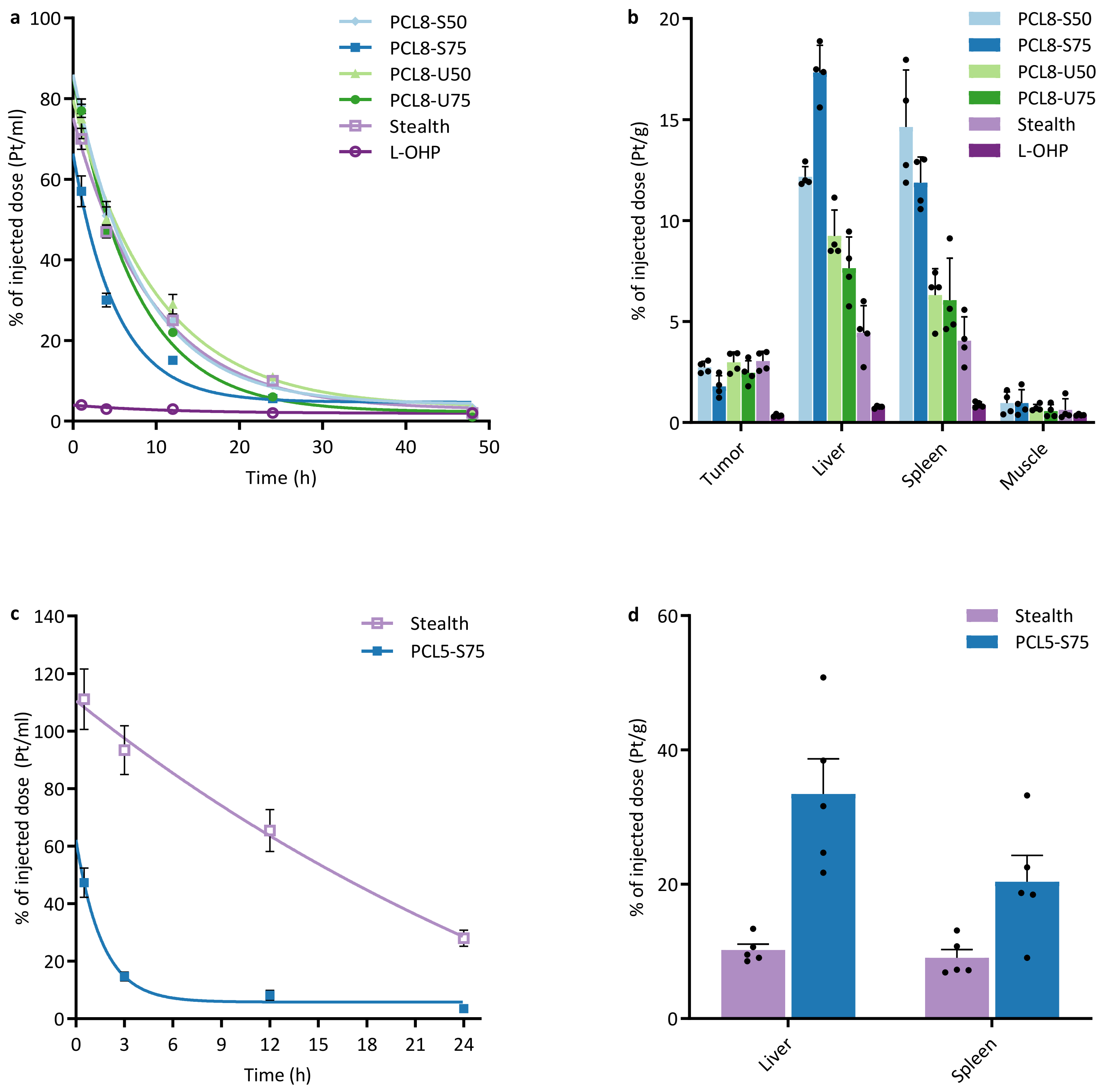

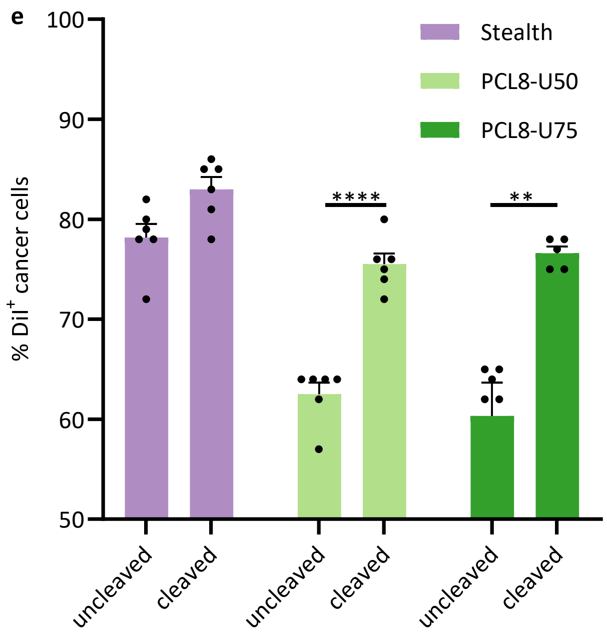

Fig. S1

Pharmacokinetics, biodistribution and uptake of cleaved liposomes

NMRI mice were injected with 6 mg/kg of Stealth L-OHP or SS75-PCL5-L-OHP. Blood samples were taken at the indicated time points and transferred to heparin-PBS containing tubes. 24 h after injection the mice were sacrificed and samples from liver and spleen were dissected. Samples were acid digested, diluted and subjected to ICP-MS measurements (a and b).

NMRI mice carrying FaDu tumors were injected with 6 mg/kg L-OHP, Stealth L-OHP, SS50-PCL8-L-OHP, SS75-PCL8-L-OHP, UU50-PCL8-L-OHP and UU75-PCL8-L-OHP liposomes. Blood samples were taken at the indicated time points and transferred to heparin-PBS containing tubes. 48 h after injection the mice were sacrificed and samples from tumor, liver, spleen and muscle were dissected. All samples were acid digested and platinum contents in blood and tissues were measured using ICP-MS (b and c).

CT26 tumors were enzymatically digested and 1x106 cells/mL in RPMI with 10% FBS were incubated with liposomes for 2 hours at 37 C°. The cleaved liposomes had been treated with or without thermolysin o/n at 37 C°. The liposomes were formulated with a DiI fluorophore, which can be detected using flow cytometry (e).

Fig. S2. Table 1. Typical characteristics of liposomes used in the studies.

Formulation	Size (nm)	PDI	Zeta potential (mV)	Lipid concentration (mM)	L-OHP conc. (mg/ml)
DSPC/Chol/DSTAP/Chol2-PCL-001. 55/32/5/8	103 ± 1	0.02	-10.8 ± 2.1	18	1.25
DSPC/Chol/DSTAP/ Chol2-PCL-001. 52.5/32/7.5/8.	106 ± 1	0.02	-7.7 ± 1.5	19	1.25
POPC/Chol/DOTAP/Chol2-PCL-001. 55/32/5/8	100 ± 1	0.01	-10.8 ± 2.7	18	1.10
POPC/Chol/DOTAP/ Chol2-PCL-001. 52.5/32/7.5/8	102 ± 1	0.05	-11.7 ± 1.9	28	0.85
DSPC/Chol/DSPE-PEG2000. 55/40/5	106 ± 1	0.02	-10.2 ± 1.3	22	0.72
DSPC/Chol/DSTAP/ Chol2-PCL-001. 52.5/35/7.5/5	127 ± 1	0.06	-10.8 ± 1.3	31	0.90

Fig. S2. Table 2. Characteristics and long-term stability of PCL8-U75.

Formulation	Size (nm)	PDI	Zeta potential (mV)	L-OHP conc. (mg/ml)	Encapsulation grade (%)
PCL8-U75 after preparation	108 ± 1	0.05	-9.8 ± 1.2	1.1	99.6
1 month	106 ± 1	0.03	-9.2 ± 2.3	1.1	99.4
Six months	112 ± 1	0.03	-10.3 ± 1.8	1.1	99.8

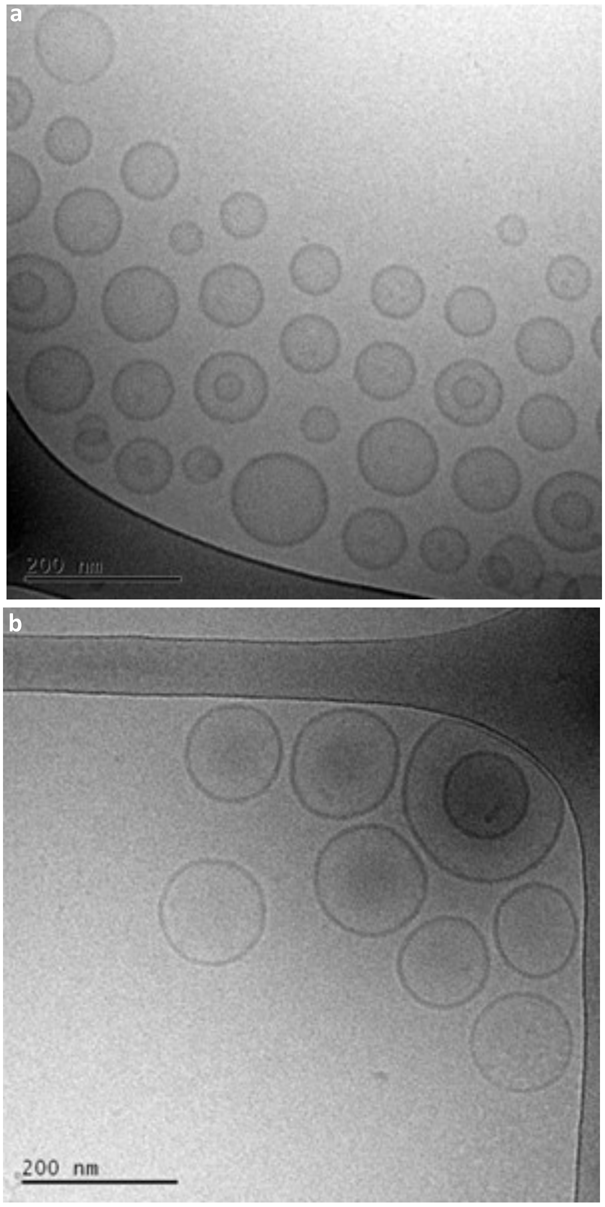

Fig. S3

Cryo-TEM of protease-sensitive liposomes. PCL8-U75 L-OHP liposomes were prepared by hydration of freeze dried lipids and subjected to Cryo-TEM (a). PCL8-S75 L-OHP liposomes were prepared by hydration of freeze dried lipids and subjected to Cryo-TEM (b).

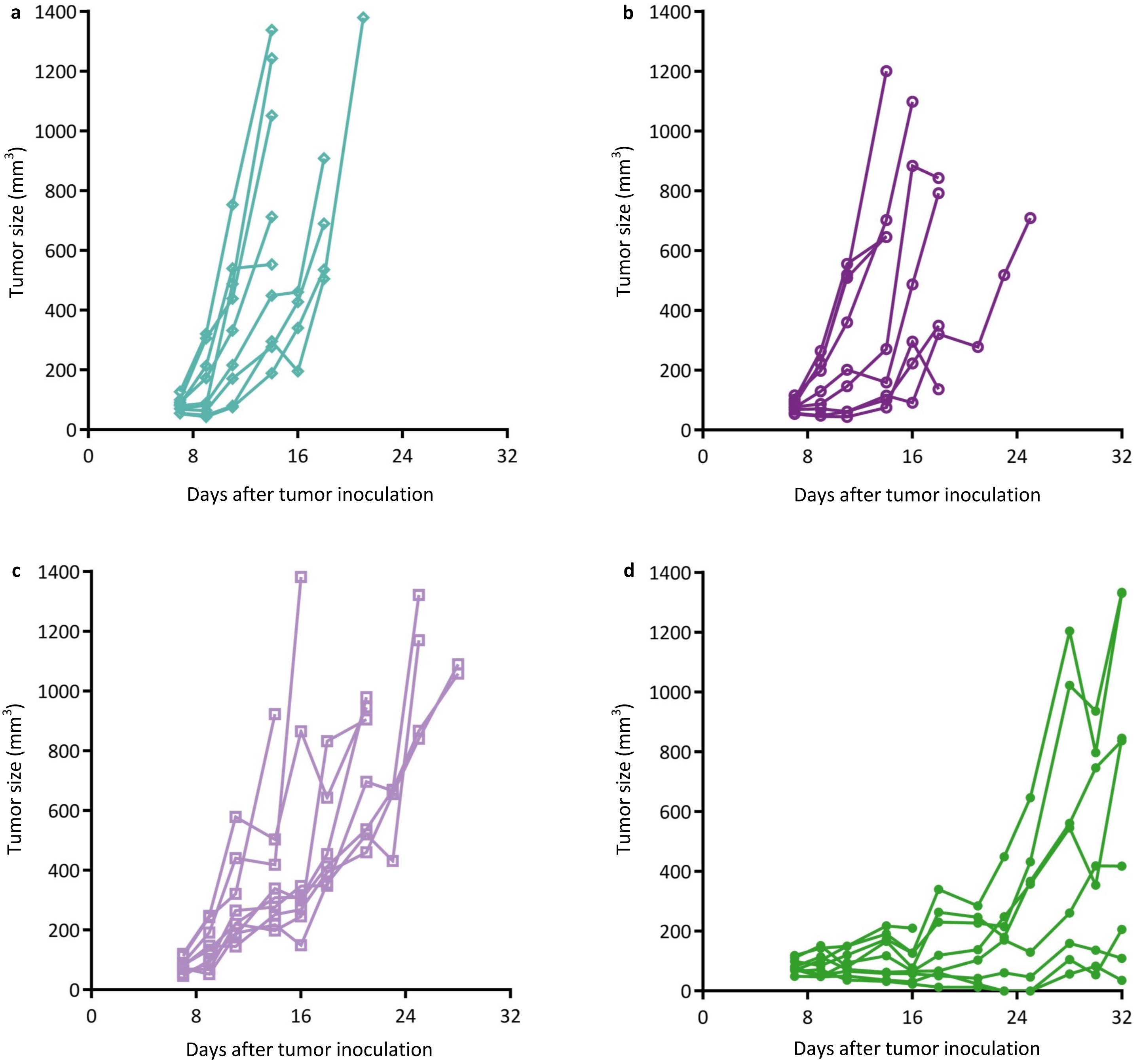

Fig. S4
Tumor sizes from individual mice generating the mean shown in Fig. 3a from B16.F10 inoculated mice. (a) Control, (b) L-OHP 8 mg/kg, (c) Stealth 8 mg/kg, (d) PCL8-U75 L-OHP 8 mg/kg.

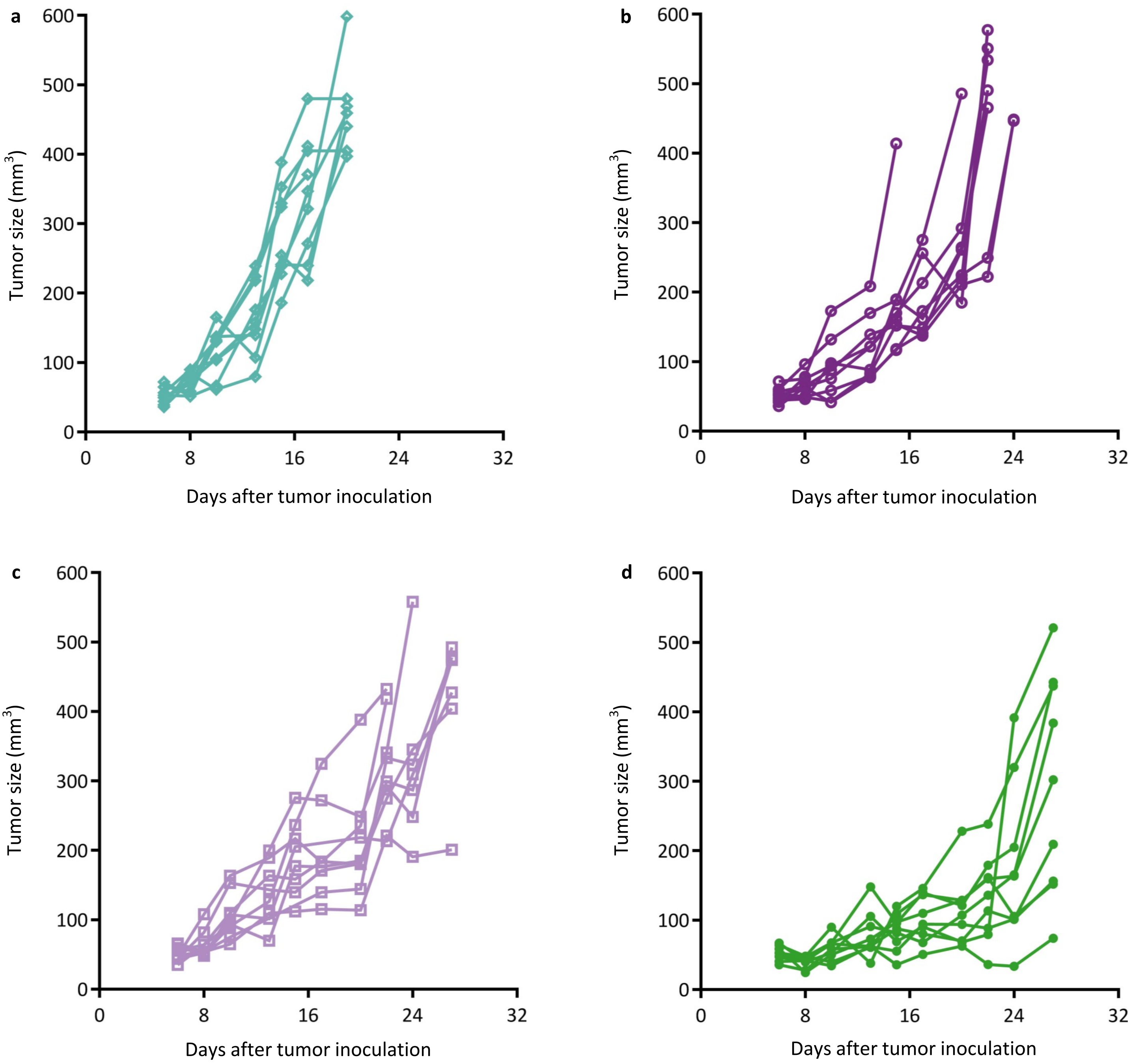

Fig. S5
Tumor sizes from individual mice generating the mean shown in Fig. 3b from 4T1 inoculated mice. (a) Control, (b) L-OHP 8 mg/kg, (c) Stealth 8 mg/kg, (d) PCL8-U75 L-OHP 8 mg/kg.

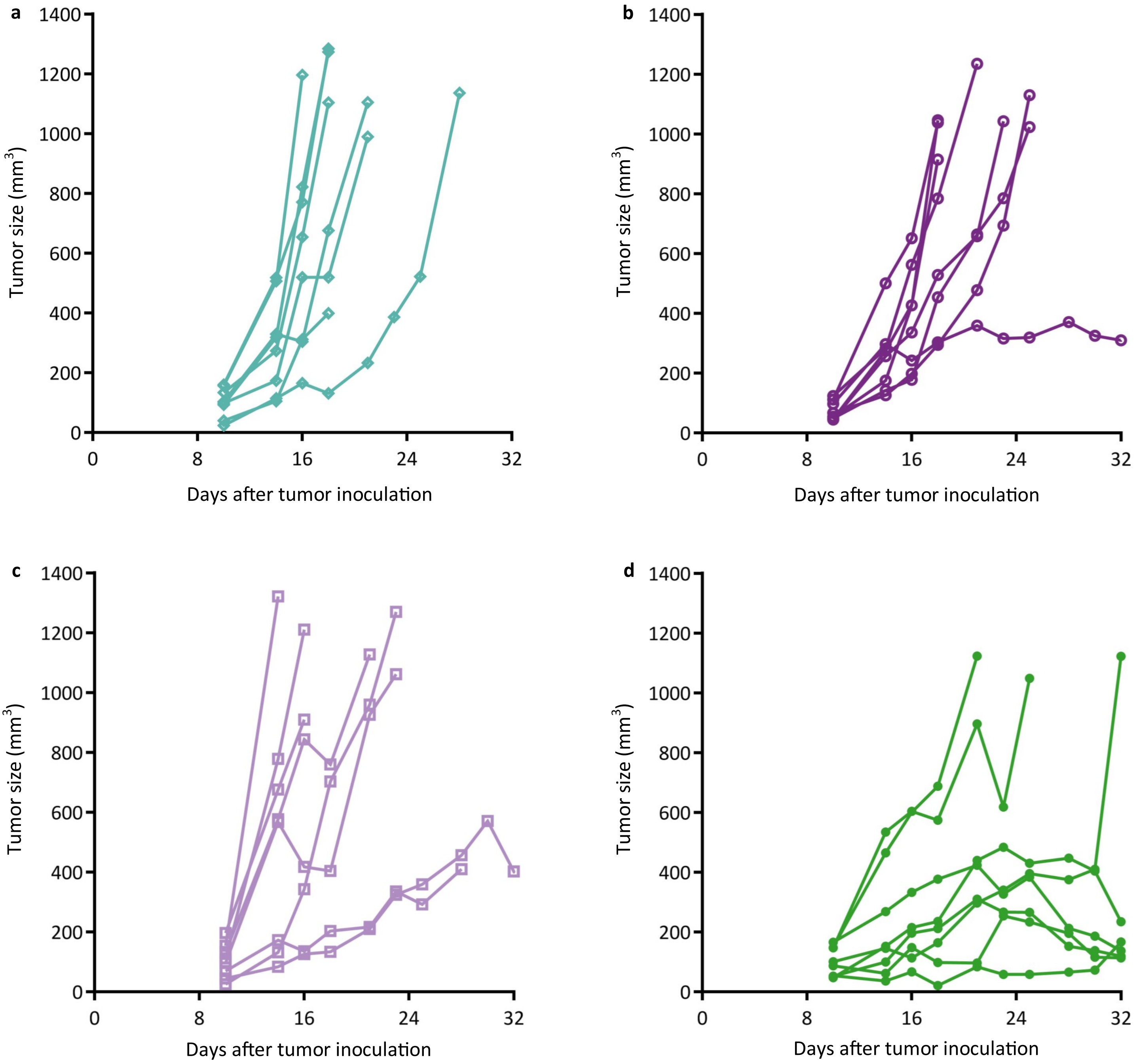

Fig. S6

Tumor sizes from individual mice generating the mean shown in Fig. 3c from CT26 inoculated mice. (a) Control, (b) L-OHP 8 mg/kg, (c) Stealth 8 mg/kg, (d) PCL8-U75 L-OHP 8 mg/kg.

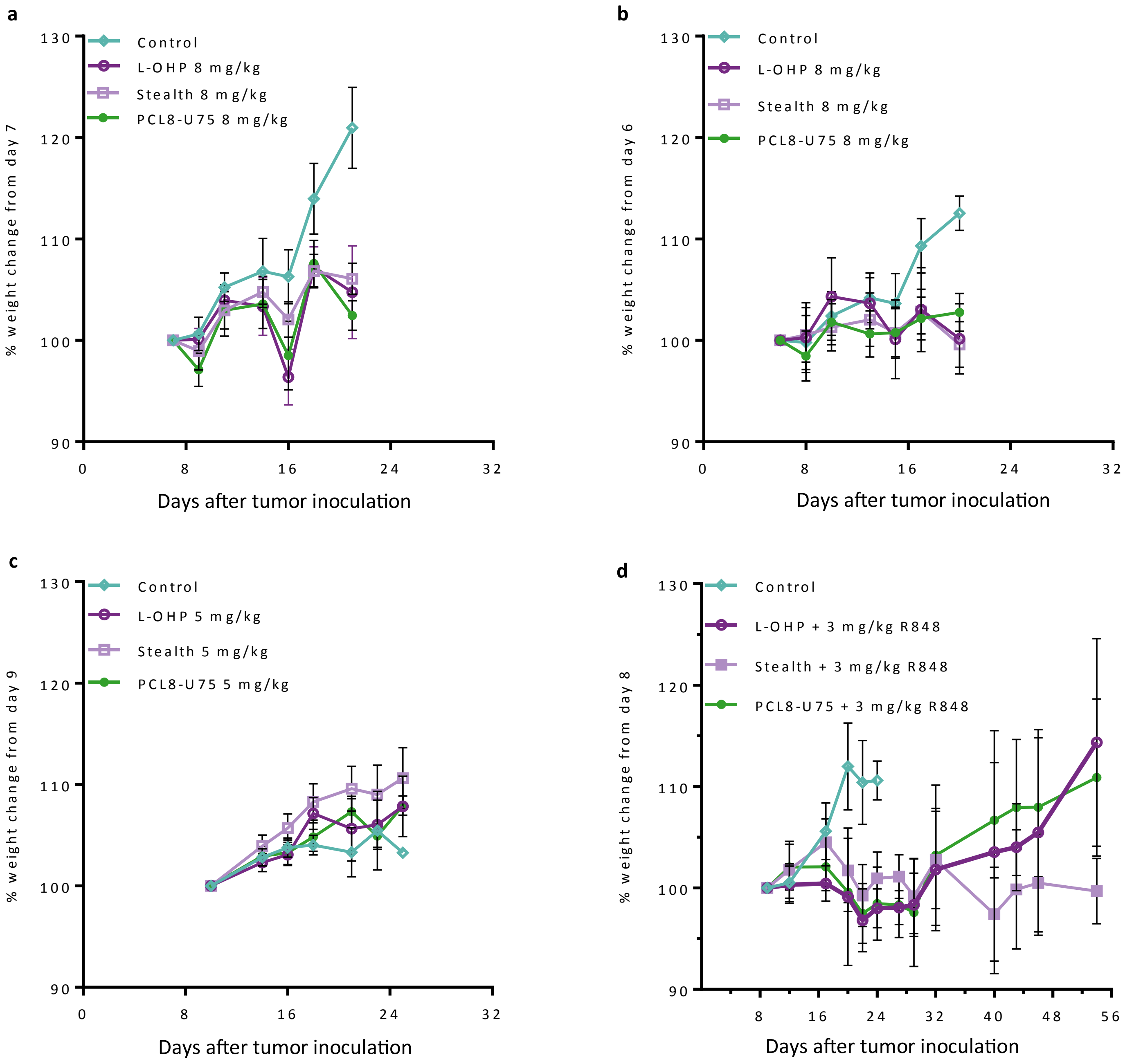

Fig. S7

Average change in body weight compared to first day of injection. (a) mice in Fig. 3a, (b) Fig. 3b, (c) Fig. 3c and (d) Fig. 4a.

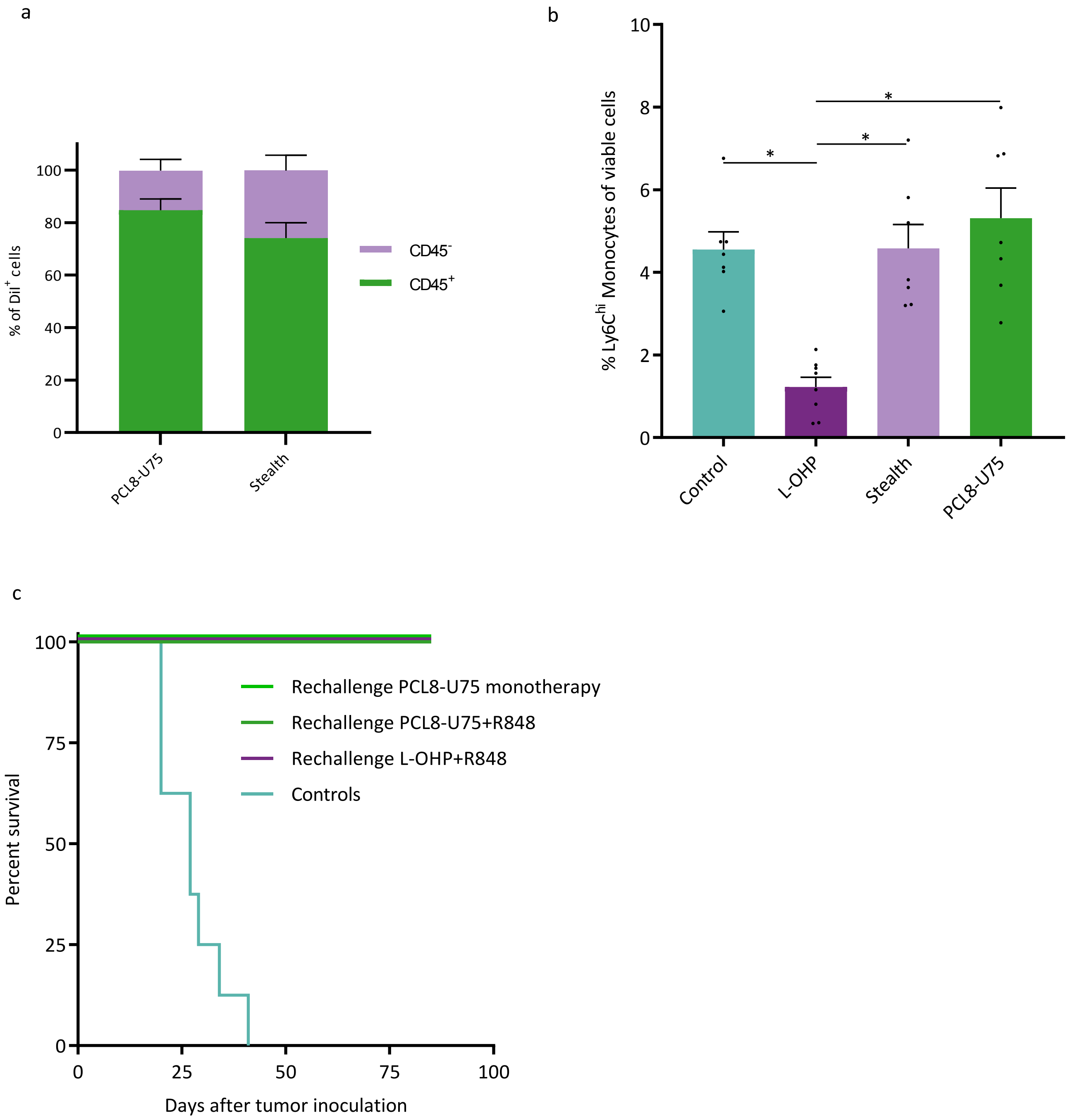

Fig. S8

Liposomes accumulates predominantly in CD45+ cells and free L-OHP depletes inflammatory monocytes in circulation

Interstacked Bar plot showing the distribution of CD45+ or CD45- of all DiI+ cells. PCL8-U75 or Stealth liposomes were formulated with DiI and injected i.v. Analysis with flow cytometry was performed after 24 hours. The cells were gated time/fsc singlets/scatter/viable/DiI+. The gate was set from a full stained untreated control (a).

Bar plot showing the percentage of systemic inflammatory Monocytes (CD11b+ Ly6Glo Ly6Chi) from whole blood 48 hours after third treatment of free L-OHP, stealth L-OHP or L-OHP PCL8-U75. Significant difference $p<0.05$. n=7-8. (b)

Kaplan-Meier plot with survival data of re-challenged complete responders from figure 3 and 4. Treated mice that survived beyond day 100 were re-challenged with 300.000 CT26 cells in the opposite flank and compared to inoculated naïve mice (c). PCL8-U75 monotherapy n=1, PCL8-U75 + R848 n=10, L-OHP + R848 n=1, naïve controls n=8.

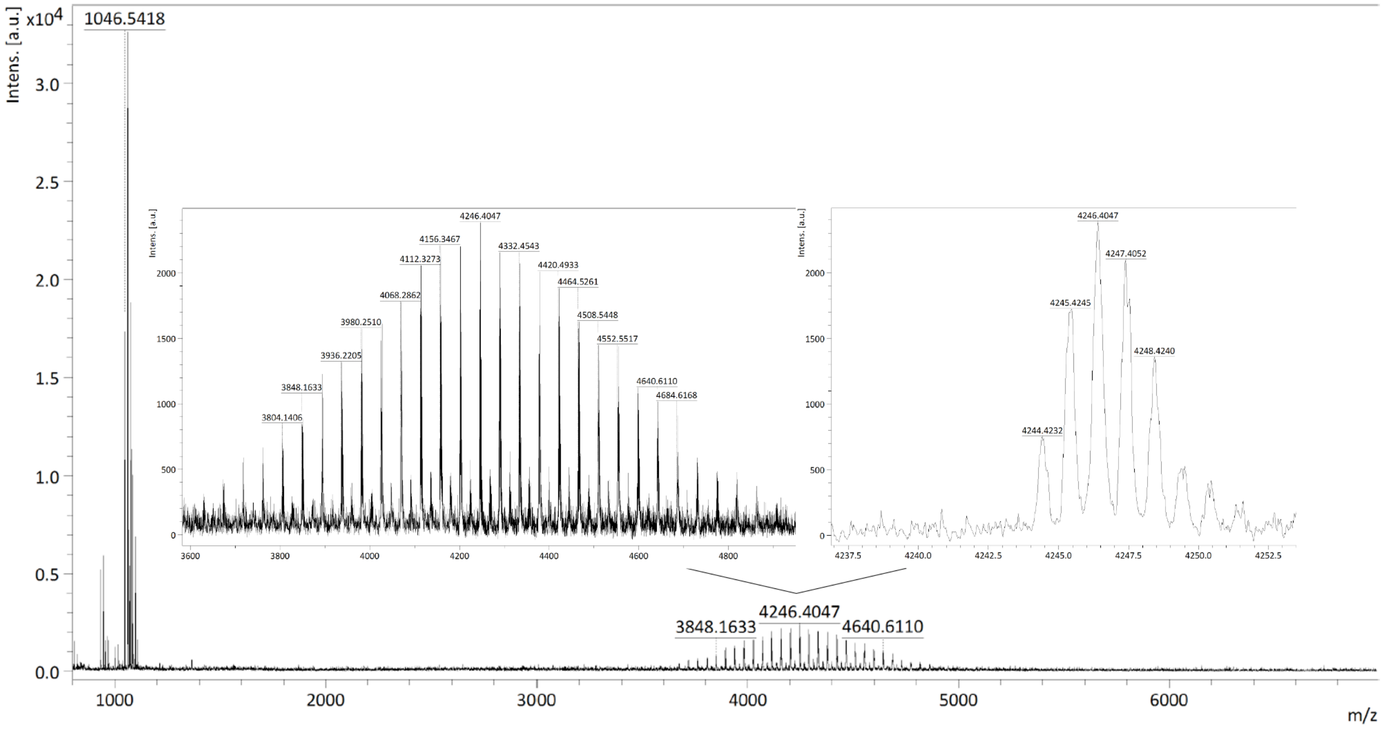

Fig. S9

MALDI-TOF MS spectrum of PCL with as DHB-matrix. Angiotensin II is used as internal calibration standard: Calc. mass ($C_{50}H_{72}N_{13}O_{12}$, [M+H]+) 1046.5418. PCL: Calc. mass ($C_{199}H_{356}N_{19}O_{77}$, [M+H]+) 4244.4520; found mass [M+H]+ 4244.4232 ± n×44.0 (Mass accuracy: 6.8 ppm)

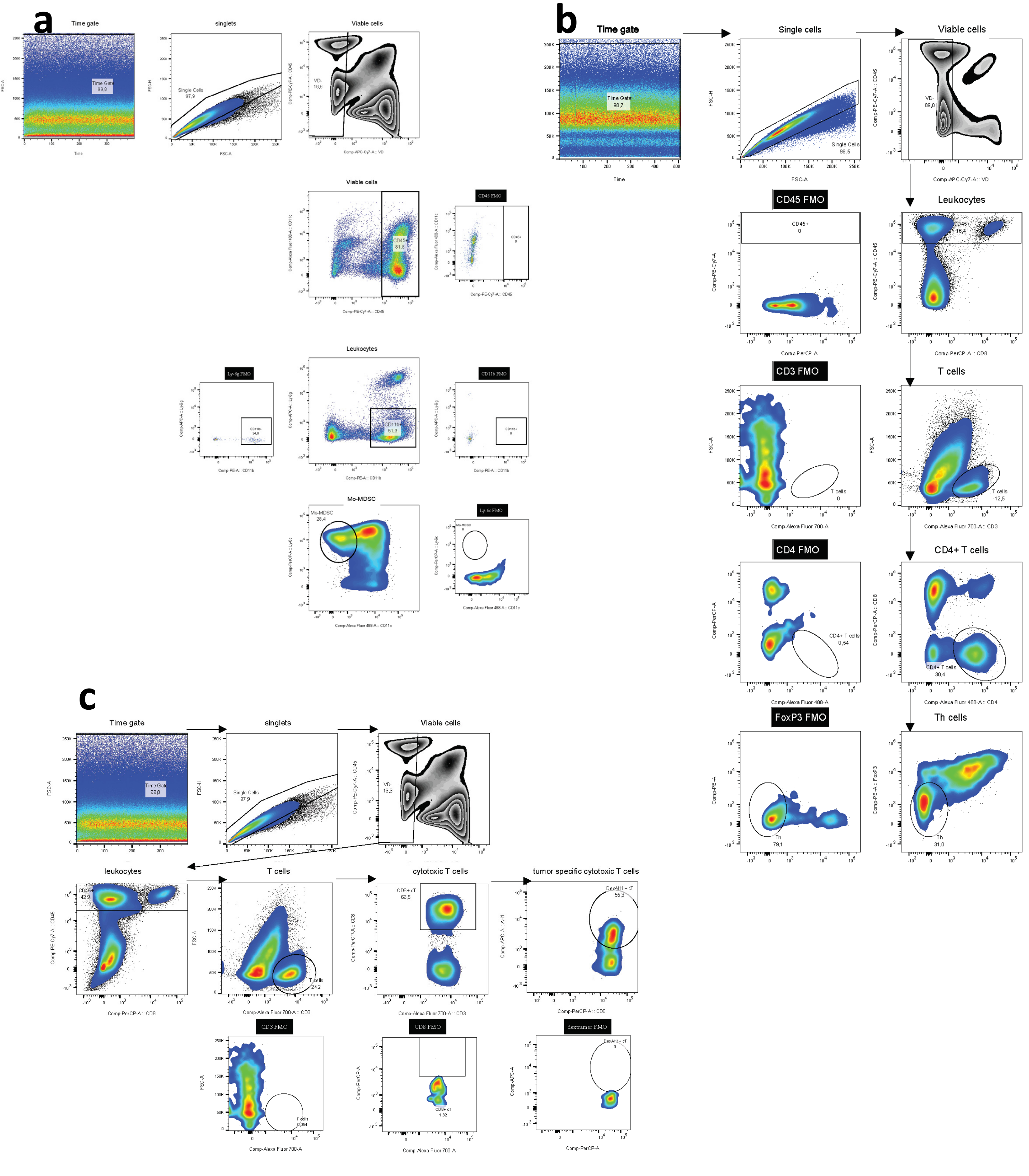

Fig. S10

Gating strategy of monocytic myeloid derived cells (Mo-MDSCs) (a). Gating strategy of T helper cells (Th) (b). Gating strategy of AH-1 (SPSYVYHQF) dextramer^{+} cytotoxic T cells (c).
